# Une présentation atypique de la maladie cœliaque: l'occlusion de la veine centrale de la rétine

**DOI:** 10.11604/pamj.2015.22.300.8196

**Published:** 2015-11-25

**Authors:** Taieb Jomni, Syrine Bellakhal, Maher Abouda, Imene Abdelaali, Hédi Douggui

**Affiliations:** 1 Unité de Gastroentérologie, Hôpital des Forces de Sécurité Intérieure, La Marsa, Tunisie

**Keywords:** Maladie cœliaque, thrombose, régime sans gluten, celiac disease, thrombosis, gluten-free diet

## Abstract

Parmi les complications thrombotiques de la maladie cœliaque l'occlusion de la veine centrale de la rétine a été exceptionnellement décrite. Nous rapportons l'observation d'une patiente âgée de 27 ans chez qui le diagnostic de maladie cœliaque a été porté dans le cadre du bilan étiologique d'une occlusion de la veine centrale de la rétine. L'interrogatoire ne révélait pas de diarrhée chronique ou de douleurs abdominales. La présence d'un amaigrissement, d'une anémie ferriprive et d'une hypocholestérolémie ont permis l'orientation vers la maladie cœliaque. La positivité des anticorps anti endomysium et la biopsie duodénale montrant l'atrophie villositaire confirmaient ce diagnostic. Le régime sans gluten associé à un traitement par aspirine avait partiellement amélioré l'acuité visuelle chez notre patiente. Cette présentation atypique de la maladie cœliaque souligne la diversité des manifestations extra-digestives au cours de cette maladie et l'intérêt de penser à la maladie cœliaque même lorsque ces manifestations sont inaugurales.

## Introduction

La maladie cœliaque (MC) est une maladie auto-immune provoquée par un antigène alimentaire: la gliadine du gluten. Les formes classiques révélées par la triade diarrhée-douleurs abdominales-malabsorption ne représentent que 10 à 20% des cas et la présentation clinique est souvent polymorphe. Les signes extra-digestifs peuvent être au premier plan et constituer la circonstance de découverte de la MC. La MC confère un sur-risque de développer une thrombose veineuse profonde et la thrombose est souvent de siège inhabituel (veines sus-hépatiques, tronc porte, veine splénique, veine mésentérique, thrombose veineuse cérébrale). La thrombose de la veine centrale de la rétine a été rarement rapportée au cours de la MC, elle est exceptionnellement révélatrice de la MC.

## Patient et observation

**Figure 1:**
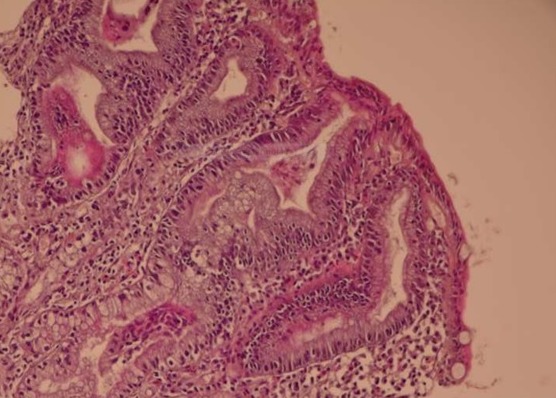
Biopsie duodénale de notre patiente montrant l'atrophie des villosités duodénales avec augmentation des lymphocytes intra épithéliaux

une femme âgée de 27 ans sans facteurs de risque de maladie veineuse thromboembolique, était adressée pour bilan étiologique d′une occlusion de la veine centrale de la rétine de l′œil droit révélée par une baisse brutale de l′acuité visuelle et confirmée par l′angiographie rétinienne à la fluorescéine. L′interrogatoire révélait un amaigrissement depuis 2 mois sans douleurs abdominales, ni diarrhée. L′examen notait une pâleur cutanéo-muqueuse et un Index de Masse Corporelle (IMC) à 17 kg/m^2^. Le bilan biologique révélait une anémie hypochrome microcytaire à 9 g/dl, une ferritinémie à 2 ng/ml et une hypocholestérolémie à 0,8g/l. La recherche des AAN, des Anti-β2Gp1 ainsi que des anti-cardiolipines était négative. Les anticorps endomysium étaient fortement positifs et le bilan de thrombophilie concluait à un déficit en proteine C et S. La fibroscopie digestive montrait des valvules conniventes espacées et de hauteur diminuée et les biopsies duodénales confirmaient l′atrophie villositaire grade 3 compatible avec le diagnostic de MC (Figure 1). La patiente était traitée par aspirine à la dose de 100 mg par jour associée au régime sans gluten, l′évolution était marquée par la prise de poids avec une amélioration partielle de l′acuité visuelle.

## Discussion

Le siège atypique de la thrombose, l'existence d'un syndrome de malabsorption et d'une anémie ferriprive chez notre patiente ont permis l′orientation vers le diagnostic de MC. Les mécanismes de la thrombose au cours de la MC sont multiples: le défaut d′absorption de la vitamine K qui accompagne le syndrome de malabsorption est responsable des déficits en protéine C et S ou en antithrombine [1], les carences en vitamines B12 et en folates et l′hyperhomocysteinémie qui en découle constituent un facteur favorisant la thrombose [2], les patients atteints de MC ont une plus forte prévalence de positivité des anticorps anti-phopholipides [3]. En dehors de ces mécanismes, l′occlusion de la veine centrale de la rétine serait favorisée par l′hyperviscosité sanguine elle même conséquence de la diarrhée chronique et du syndrome de malabsorption [4]. Dans la littérature seulement quelques cas d′occlusion de la veine centrale de la rétine associés à la MC ont été rapportés [4, 5].

## Conclusion

La réversibilité des carences vitaminiques et des déficits en protéine C et S sous régime sans gluten rend compte de l'importance de faire le diagnostic MC dans le cadre du bilan étiologique d′une occlusion de la veine centrale de la rétine. Bien que atypique cette présentation rare de la MC doit être présente à l′esprit du praticien. La collaboration entre ophtalmologiste, gastroentérologue et interniste augmente la rentabilité de l′enquête étiologique.
